# Traumatic Endothelial Corneal Rings

**DOI:** 10.18502/jovr.v15i4.7797

**Published:** 2020-10-25

**Authors:** Hossein Mohammad-Rabei, Amir A. Azari, Amir Arabi

**Affiliations:** ^1^Ophthalmic Research Center, Shahid Beheshti University of Medical Sciences, Torfe Medical Center, Tehran, Iran

##  PRESENTATION

A 49-year-old woman sustained corneal injury after explosion of a water heating system at home. On biomicroscopic examination, multiple foreign bodies were seen on the epithelial surface of the cornea without causing any damage to the stromal tissue. Further examination revealed multiple annular ring-shaped opacities at the level of the corneal endothelium in both eyes.

**Figure 1:**
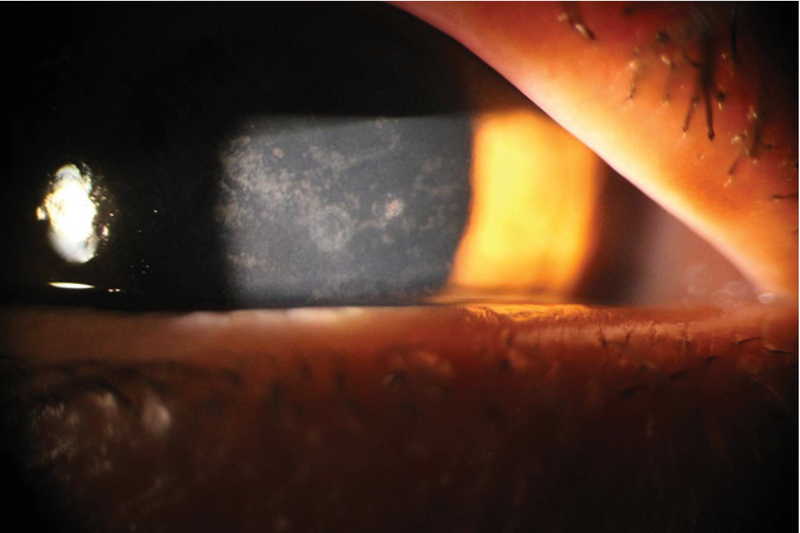
Multiple small epithelial defects secondary to several high-velocity projectiles. Note the central endothelial ring opacity.

**Figure 2:**
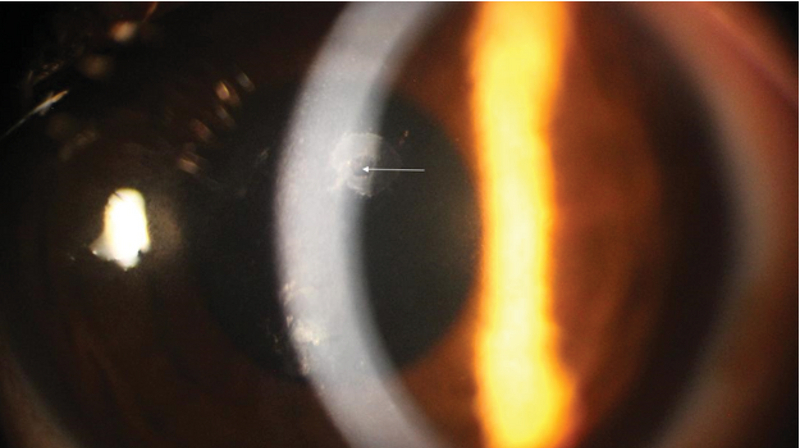
At higher magnification, central healthy endothelial cells are surrounded by a well-defined ring shape opacity.

**Figure 3:**
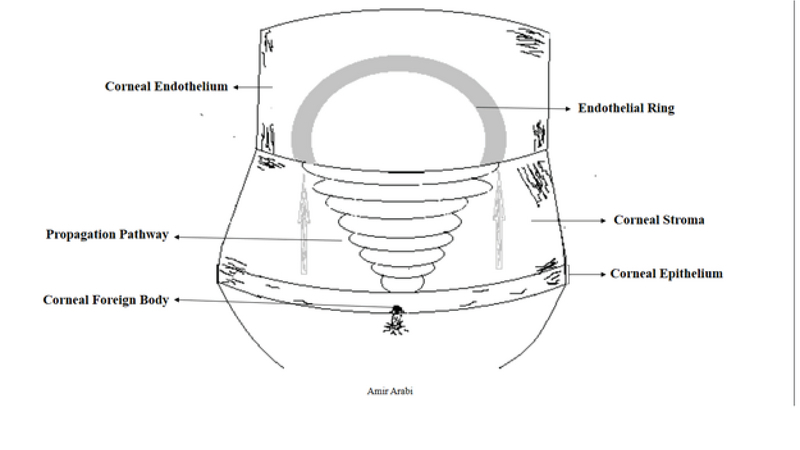
A schematic view for propagation of the force from corneal epithelial surface to the endothelial layer.

##  DISCUSSION

Traumatic corneal endothelial rings are a rare consequence of small high-velocity foreign body projectiles hitting the cornea most commonly from blast injuries.^[1]^ In patients with endothelial corneal rings, the injury to the endothelial cells results from damage caused by mechanical stretching of the cornea. Corneal stretching causes axial displacement of Descemet's membrane, producing radial tension transmitted further posteriorly damaging the delicate endothelial cells.^[2]^ The ring-shaped disruption of the endothelial cells likely forms as a result of a bell-shaped propagation of shock waves produced by trauma. As these waves travel through Bowman's membrane, corneal stroma, Descemet's membrane and all the way to the endothelium, they impact the peripheral endothelial cells most severely while leaving the central endothelial cells relatively unaffected.

Maloney et al used specular microscopy in two patients with traumatic endothelial rings. Based on their findings, they reported endothelial cell loss in all cases with traumatic endothelial rings.^[3]^

Endothelial rings appear soon after injury and tend to disappear in a just a few days. Sung Jin Kim et al reported that the mean duration for the presence of rings was 4.6 days on average.^[2]^ When the total number of endothelial cells lost is small, as evident when only a few corneal endothelial rings are present, one should expect an unremarkable clinical course; however, with more severe and extensive injuries, ophthalmologists should be wary of potential corneal decompensation if further endothelial cell loss is anticipated form future intraocular surgeries and procedures.^[4]^

In our case, traumatic corneal endothelial rings were clinically visible immediately after the injury, and resolved completely within one week without any specific treatment.

##  Financial Support and Sponsorship

Nil.

##  Conflicts of Interest

There are no conflicts of interest.
